# Effect of Digital Self-Monitoring on Patient Engagement and Clinical Outcomes in Severe Asthma: A Randomized Controlled Pilot Study

**DOI:** 10.3390/medicina62020368

**Published:** 2026-02-12

**Authors:** Norbert Wellmann, Versavia Maria Ancusa, Monica Steluta Marc, Ana Adriana Trusculescu, Ioana Ciortea, Flavia Gabriela Martis, Pescaru Andrei, Andreea Roxana Durdan, Ovidiu Fira-Mladinescu

**Affiliations:** 1 Pulmonology University Clinic, Clinical Hospital of Infectious Diseases and Pneumophthisiology “Dr. Victor Babeș”, 13 Gheorghe Adam Street, RO-300310 Timisoara, Romania; norbert.wellmann@umft.ro (N.W.); flavia.martis@umft.ro (F.G.M.); andreea.durdan@rezident.umft.ro (A.R.D.); mladinescu@umft.ro (O.F.-M.); 2 Center for Research and Innovation in Personalized Medicine of Respiratory Diseases (CRIPMRD), “Victor Babeș”, Pneumology University Clinic, University of Medicine and Pharmacy Timișoara, Eftimie Murgu Square 2, RO-300041 Timisoara, Romania; ioana.ciortea@umft.ro; 3 Doctoral School, “Victor Babeș” University of Medicine and Pharmacy Timisoara, Eftimie Murgu Square 2, RO-300041 Timisoara, Romania; andrei.pescaru@umft.ro; 4 Department of Computer and Information Technology, Automation and Computers Faculty, “Politehnica” University of Timișoara, Vasile Pârvan Blvd, No. 2, RO-300223 Timisoara, Romania; versavia.ancusa@upt.ro; 5 Department of Biology and Life Sciences, “Vasile Goldiș” University, 94 Revoluției Boulevard, RO-310002 Arad, Romania; 6 Obstetrics and Gynecology Unit II, Emergency Clinical Municipal Hospital, RO-300079 Timisoara, Romania

**Keywords:** severe asthma, telemedicine, digital health, home spirometry, adherence, patient engagement, reminders, e-health

## Abstract

*Background and Objectives*: Severe asthma poses significant clinical and economic burdens, with adherence to monitoring and treatment remaining a challenge despite biologic therapies. This pilot study aimed to evaluate the feasibility of telemedicine-based home monitoring using the AioCare system in patients with severe asthma and to determine if weekly reminder messages improved adherence compared to standard monitoring. *Materials and Methods*: In this prospective, single-center randomized controlled pilot study, 30 adults with severe asthma were assigned to either a reminder group (weekly SMS or in-app messages) or a control group without reminders. All participants performed weekly home spirometry for 12 weeks using the AioCare system. Lung function parameters, Asthma Control Test (ACT) scores, adherence to monitoring, and patient satisfaction were assessed. Longitudinal data were analyzed using mixed-effects and generalized estimating equation models. *Results*: Adherence to home monitoring was significantly higher in the reminder group (11.47 ± 0.92 vs. 9.13 ± 3.16 sessions; *p* = 0.044). Overall, patient satisfaction was higher in the intervention group (*p* = 0.0044), with universal endorsement of the reminders and perceived educational benefit. No significant between-group differences were observed in lung function parameters. ACT scores showed a favorable trend in both groups, with a medium between-group effect size favoring the intervention (d = 0.42), although this did not reach statistical significance. *Conclusions*: Home monitoring with reminders is feasible, safe, and enhances adherence and satisfaction in severe asthma, although it did not significantly affect short-term changes in lung function or symptom control. Larger, longer-term studies are warranted to determine whether these engagement benefits translate into improved long-term clinical outcomes.

## 1. Introduction

Asthma is a heterogeneous obstructive respiratory condition that is increasingly acknowledged as a major global health issue, especially in urban areas. Due to progress in airway immunobiology, two common inflammatory phenotypes have been identified: type 2 (T2)-high and T2-low. Different biological processes control each phenotype, which helps doctors come up with personalized treatment plans. Less than 10% of people with asthma have severe cases, but this small group has the most clinical and financial problems because they have frequent flare-ups, a lower quality of life, and a lot of resistance to standard treatments [1,2,3].

Intricate inflammatory and redox-mediated pathways, according to new evidence, influence severe respiratory diseases and may cause clinical instability if not meticulously monitored. These processes highlight the need for continuous assessment and timely intervention, as insufficient follow-up and persistent inflammatory activity in other severe pulmonary conditions have been associated with poorer outcomes [4,5].

By lowering inflammation, exacerbations, and oral corticosteroid reliance, biologic treatments that target IgE, IL-5, IL-4/IL-13 pathways, or TSLP have revolutionized the treatment of severe asthma. However, real-world adherence, patient engagement, and ongoing assessment—areas where traditional clinic-based follow-up is limited—are also necessary for the best disease control [1,2,3].

Telemedicine and digital health technologies, such as portable home spirometry systems, present innovative opportunities for the remote monitoring of lung function, symptom trends, and medication response in severe asthma.

By enabling continuous assessment outside the clinical setting, these tools may facilitate earlier identification of loss of control, support self-management, and complement advanced biologic therapies. Remote monitoring has therefore become an increasingly explored strategy in the management of chronic respiratory disease. However, real-world adherence to digital monitoring remains inconsistent, representing a major barrier to the effectiveness of telemedicine interventions. This highlights the need to better understand patient engagement and to identify simple, scalable strategies that support sustained participation in home monitoring programs [6,7,8,9,10,11,12,13].

This study aimed to assess adherence to telemedicine-based home monitoring using the AioCare digital respiratory system in patients with severe asthma over a three-month period, and to determine whether weekly reminder messages sent via SMS or the AioCare platform enhanced adherence compared with standard remote monitoring. Importantly, this investigation was designed primarily as a feasibility and engagement study, with adherence as the main outcome, while clinical parameters were included as exploratory secondary measures.

## 2. Materials and Methods

### 2.1. Study Design and Setting

This prospective, single-center, randomized controlled pilot study enrolled 30 adult patients diagnosed with severe asthma who initiated home-based spirometry monitoring using AioCare portable spirometer devices (HealthUp S.A., Warsaw, Poland) and were followed over a three-month period. The study was retrospectively registered at ClinicalTrials.gov (Identifier: NCT07393984) on 25 January 2026, after participant enrollment had begun. Prospective registration was not performed because the study was initially conceived as a pilot feasibility and engagement study focusing primarily on adherence, usability, and patient-reported outcomes rather than therapeutic efficacy. Retrospective registration was subsequently completed to ensure transparency and public accessibility of the protocol; however, this represents a methodological limitation, as retrospective registration does not fully align with best clinical trial practices and may increase the risk of reporting bias. The study was designed to assess feasibility and to generate preliminary effect-size estimates to inform future adequately powered trials. Patients were randomized into two groups: an intervention group (*n* = 15) receiving weekly reminder messages to perform measurements, and a control group (*n* = 15) without reminders. The study was conducted at “Victor Babeș” University Hospital in Timișoara, Romania, between October 2024 and October 2025.

### 2.2. Ethical Approval

Both the hospital ethics committee (approval No. 8528/25.09.2024) and the university ethics committee (approval No. 49/01.10.2024) approved this study. Prior to inclusion, written informed consent was obtained from each participant.

### 2.3. Participants

Additional inclusion criteria were diagnosis of severe asthma according to GINA guidelines, age 18 years or older, ability to perform spirometry maneuvers correctly according to ATS/ERS standards after training, access to a smartphone compatible with the AioCare application, willingness to participate in weekly home-based spirometry monitoring and telemonitoring over three months, and stable maintenance therapy for at least four weeks prior to enrollment. Exclusion criteria included age under 18 years, diagnosis of other significant respiratory diseases (chronic obstructive pulmonary disease, interstitial lung disease, bronchiectasis), recent asthma exacerbation requiring hospitalization or systemic corticosteroids within the past four weeks, severe psychiatric or cognitive impairment that could interfere with adherence to the monitoring protocol or ability to use the device independently, inability to perform acceptable spirometry maneuvers despite training, lack of smartphone or internet access required for AioCare telemonitoring, pregnancy or breastfeeding, and refusal to provide informed consent.

### 2.4. Data Collection and Management

Data were systematically collected and organized using Microsoft Excel, including demographic (age, sex, BMI), clinical (biological therapy, smoking status), recorded at baseline. Patient-reported outcomes, including the Asthma Control Test (ACT), were assessed at baseline and at the three-month follow-up period. Respiratory parameters collected through weekly home monitoring included spirometry variables (FVC, FEV_1_, FEV_1_/FVC, FEF_25–75_), peak expiratory flow (PEF), oxygen saturation (SpO_2_), and heart rate.

The 10-item AioCare Satisfaction Questionnaire was administered once, at study completion. At the final study visit, participants returned the AioCare devices, and transmitted monitoring data were reviewed for completeness and integrity. To ensure data integrity and patient confidentiality, all records were anonymized using unique patient identifiers (P001–P020).

### 2.5. Randomization and Blinding

A computer-generated randomization sequence randomly allocated participants to either the intervention or control group in a 1:1 ratio. The trial was open-label, meaning that both participants and clinicians knew which group they were assigned to. There was no blinding in the outcome evaluation.

### 2.6. Remote Monitoring and Adherence Support

All participants received standard asthma care in accordance with current guidelines and began home monitoring using the AioCare system. The study intervention consisted solely of weekly reminder messages designed to support adherence to home spirometry measurements.

All patients were remotely monitored via the AioCare cloud platform, which automatically transmitted every validated home spirometry, peak expiratory flow (PEF), SpO_2_, and heart rate measurement to the clinical team in real time. The platform provided continuous information on measurement frequency, spirometry quality (according to ATS/ERS acceptability and repeatability criteria), and longitudinal trends in lung function parameters throughout the 3-month study period.

Patients were randomized 1:1 to:

Intervention (reminder) group (*n* = 15): at enrolment, each patient was assigned a single preferred reminder channel—either SMS to their registered mobile phone number or a push notification/in-app message within the AioCare mobile application—based on technical capabilities and personal preference. A short, standardized reminder was then sent every Monday morning exclusively through the chosen channel.

Control group (*n* = 15): received no reminders and performed measurements independently in accordance with the study protocol.

The content of the reminder was identical regardless of the chosen channel: Reminder: “Please perform your weekly spirometry measurement today. Thank you for your participation”.

This simple, single-channel, low-cost strategy was deliberately designed to be easily reproducible in everyday clinical practice. No telephone calls or additional human interventions were planned unless the patient reported technical problems or clinical worsening.

### 2.7. Clinical Assessments

Asthma Control: At baseline and following the three-month monitoring period, symptom control was evaluated using the Asthma Control Test (ACT). Better asthma control is indicated by higher ACT scores, which range from 5 to 25.

AioCare User Experience: At the conclusion of the three-month follow-up period, a structured questionnaire measuring usability, perceived usefulness, acceptability, satisfaction, and willingness to continue AioCare-based home monitoring was given to all patients who had completed at least the final study visit. The validated mHealth App Usability Questionnaire (MAUQ) was modified into a 10-item dichotomous (Yes/No) questionnaire [14]. To minimize respondent burden and increase completion rates among patients with severe asthma, the original Likert-scale response format was changed to Yes/No. Two supplementary questions exploring perceived clinical benefit were added to better reflect patient impressions of how AioCare influenced symptom awareness, disease management, and overall monitoring experience.

Adherence: The percentage of study weeks in which patients finished at least one valid AioCare spirometry session was used to measure adherence to telemonitoring. The percentage of weekly assessments completed over the 12-week study period was used to measure adherence: (number of completed weeks/12) × 100%. Patients were classified as adherent (≥75%), partially adherent (50–74%), or non-adherent (<50%) based on this proportion. The weekly adherence rates of the intervention and control groups were compared.

### 2.8. Statistical Analysis

Statistical analyses were performed using Python version 3.12.12, employing the following libraries: Pandas (v2.2.2), SciPy (v1.16.3), Statsmodels (v0.14.6), Scikit-learn (v1.6.1), NumPy (v2.0.2), Matplotlib (v3.10.0), and Seaborn (v0.13.2). All statistical tests were two-tailed, with a predefined significance threshold of α = 0.05.

The primary analysis followed the intention-to-treat principle. Longitudinal outcomes were analyzed using mixed-effects models with group, time, and group × time interaction as fixed effects and participant as a random effect. When model assumptions were violated, generalized estimating equations were applied.

Secondary analyses compared groups using appropriate parametric or non-parametric tests. Effect sizes were reported. Detailed statistical procedures and diagnostic testing are provided in the Supplementary Methods.

Generative artificial intelligence (GenAI) tools were used only to improve grammar, wording, and manuscript readability. They did not contribute to study design, data processing, statistical analysis, interpretation of results, or the generation of scientific content. All findings, conclusions, and responsibility for the manuscript rest with the authors.

## 3. Results

### 3.1. Participant Flow

**Figure 1:**
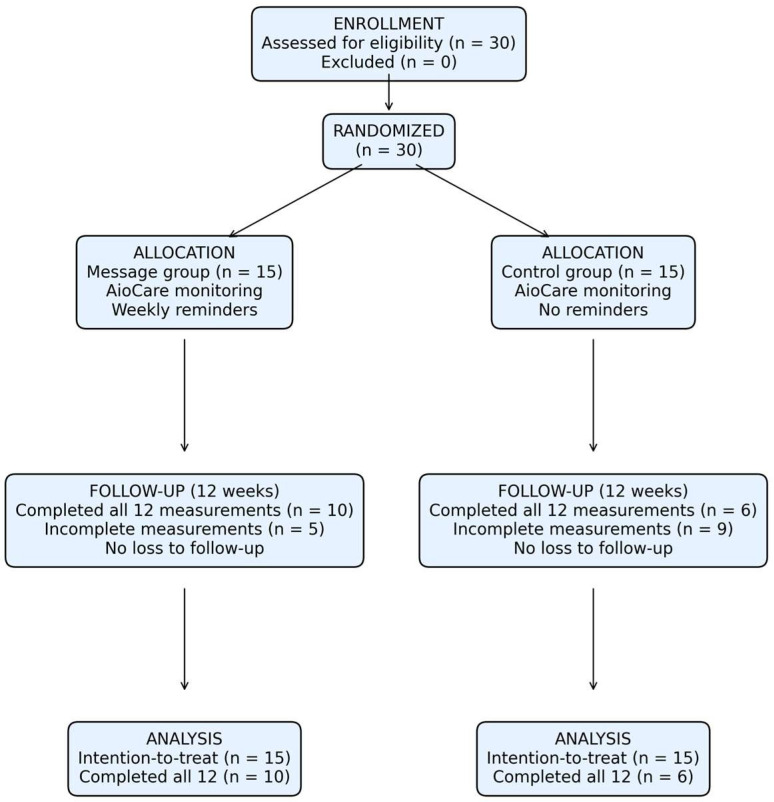
CONSORT flow diagram of participant recruitment, randomization, follow-up, and analysis.

A total of 30 patients with severe asthma were evaluated for eligibility and subsequently randomized to two groups: the intervention group (*n* = 15), received weekly reminder messages via SMS or the AioCare platform, and the control group (*n* = 15), received no reminders (Figure 1).

All participants received AioCare-based home monitoring. In the 12-week follow-up phase, 10 patients from the intervention group and 6 patients from the control group completed all 12 scheduled weekly assessments. All participants were retained without exclusion or loss of follow-up. All randomized participants were incorporated into the intention-to-treat analysis.

### 3.2. Baseline Characteristics

Baseline characteristics are presented in Table 1. The two groups were well-balanced across all measured variables. The mean age was 52.3 years in both groups, with a female predominance in the control group (66.7% vs. 40.0%, *p* = 0.272). More than half of participants in the control group were current smokers (53.3% vs. 33.3%, *p* = 0.461). All participants were receiving biological therapy for severe asthma at enrollment.

Baseline lung function indicated moderate airflow limitation, with mean FVC of 3.61 ± 1.03 L in the control group and 3.68 ± 1.18 L in the intervention group (*p* = 0.877), corresponding to 92.5 ± 20.6% and 88.0 ± 23.2% of predicted values, respectively. Mean FEV_1_ was 2.57 ± 0.91 L (81.2 ± 21.5% predicted) in the control group and 2.65 ± 1.00 L (78.0 ± 23.1% predicted) in the intervention group (*p* = 0.816). The FEV_1_/FVC ratio was reduced in both groups (control: 69.92 ± 9.60%; intervention: 70.93 ± 10.85%; *p* = 0.801), consistent with obstructive physiology.

Asthma control at baseline was suboptimal despite maximal medical therapy. Mean ACT scores were 18.7 ± 1.9 in the control group and 19.0 ± 2.0 in the intervention group (*p* = 0.642), both below the threshold for well-controlled asthma (ACT ≥20). One-third of control patients and 40% of intervention patients achieved well-controlled status at baseline, while no control patients and one intervention patient (6.7%) had poorly controlled asthma (ACT < 16). There were no significant differences between groups in the main baseline characteristic (all *p* > 0.05), confirming successful randomization.

### 3.3. Primary Outcomes—Lung Function

No significant between-group differences were observed in any lung function parameters over the 12-week monitoring period (all Group × Time *p* ≥ 0.18). Both groups showed overall stable spirometric values, with very small effect sizes and all confidence intervals crossing zero. These findings indicate similar longitudinal trajectories in objective lung function regardless of reminder exposure. Detailed longitudinal spirometry results are provided in Supplementary Table S1.

### 3.4. Adherence

**Figure 2:**
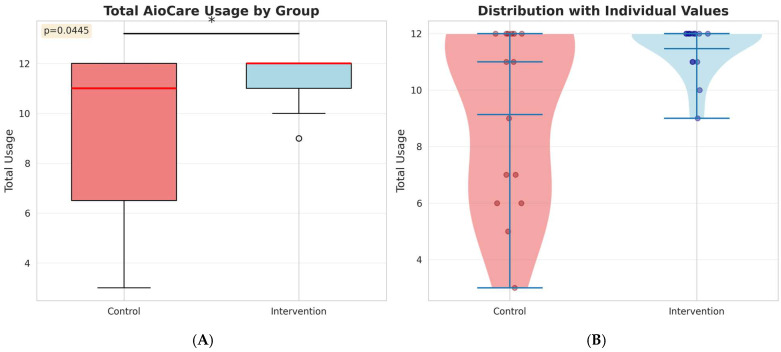
Protocol Adherence: Total AioCare Usage Over 12 Weeks. (**A**) Box plots showing median (red horizontal line), interquartile range (box), and range (whiskers) of total completed monitoring sessions. Maximum possible sessions = 12. Mann–Whitney U test: U = 67.5, *p* = 0.044, rank-biserial r = 0.40. Outliers are depicted with ◦. (**B**) Violin plots with overlaid box plots and individual patient values (dots). The intervention group (blue) shows tight clustering near maximum adherence (median = 12, IQR 11–12), while the control group (red) displays greater variability (median = 11, IQR 6.5–12). Width of violin reflects distribution density. * for *p* < 0.05 (statistically significant).

Protocol adherence, measured as the total number of completed monitoring sessions over 12 weeks (maximum possible: 12), differed significantly between groups (Figure 2). The intervention group demonstrated higher mean adherence (11.47 ± 0.92 sessions, range 9–12) compared to the control group (9.13 ± 3.16 sessions, range 3–12; Mann–Whitney U = 67.5, *p* = 0.044, rank-biserial r = 0.40). Notably, the intervention group showed substantially less variability in adherence, with all patients completing at least 9 sessions. In contrast, the control group exhibited greater heterogeneity, with adherence ranging from 3 to 12 sessions. The violin plot (Figure 2, right panel) illustrates this marked difference in adherence consistency, with the intervention group showing a narrow distribution clustered near maximum compliance, whereas the control group shows a broader distribution with several patients exhibiting poor adherence.

### 3.5. Symptom Control—ACT Scores

Asthma symptom control improved significantly in both groups over the 12-week study period (Table 2). At baseline, ACT scores were similar between groups (*p* = 0.641). Both groups demonstrated significant improvements from baseline to week 12 (control: +2.73 ± 1.58 points, *p* < 0.001; intervention: +3.40 ± 1.59 points, *p* < 0.001). The between-group difference in change scores did not reach statistical significance (t = −1.15, *p* = 0.260), although the observed effect size was medium (Cohen’s d = 0.42). Using the minimal clinically important difference threshold of 3 points, 8/15 (53.3%) control patients and 10/15 (66.7%) intervention patients achieved clinical benefit (χ^2^ = 0.139, *p* = 0.709). Although a favorable directional trend was observed, the present pilot study was not powered to detect statistically significant between-group differences in ACT scores. Therefore, these findings should be interpreted as exploratory signals rather than evidence of a treatment effect.

**Figure 3:**
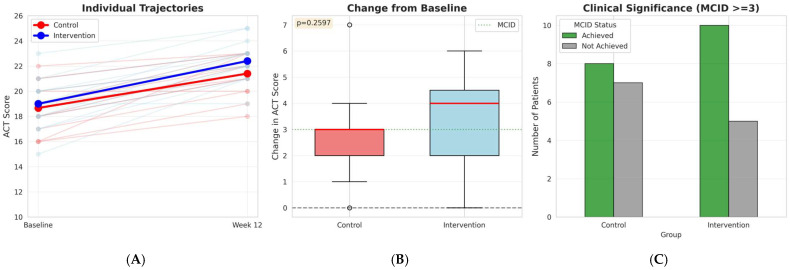
Asthma Control Test (ACT) Score Changes Over 12 Weeks. (**A**) Individual patient trajectories from baseline to week 12 for control (red) and intervention (blue) groups, with group means shown as thick lines. (**B**) Distribution of change scores (Week 12—Baseline) showing median (red horizontal line), interquartile range (box), and full range (whiskers). The dashed line represents the minimal clinically important difference (MCID) of 3 points. Between-group difference in change scores: *t* = −1.15, *p* = 0.26, Cohen’s d = 0.42. (**C**) Number of patients achieving MCID (≥3 points improvement) in each group. Green bars = achieved MCID; gray bars = did not achieve MCID (*χ*^2^ = 0.139, *p* = 0.709).

Figure 3 illustrates individual patient trajectories and the distribution of ACT score changes over the 12-week period. Panel A shows that while most patients in both groups improved, considerable interindividual variability was observed, with some patients showing minimal response. Panel B demonstrates overlapping distributions of change scores between groups, with the intervention group showing a slightly higher median improvement. Panel C shows that although a greater proportion of intervention patients achieved the MCID (66.7% vs. 53.3%), this difference was not statistically significant.

### 3.6. Patient Satisfaction

**Figure 4:**
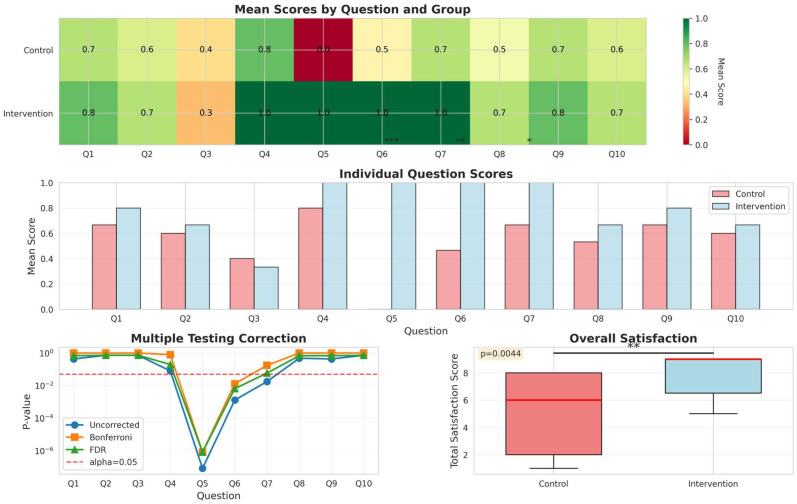
Patient Satisfaction with Monitoring System. Heatmap (**top**) and bar chart (**middle**) showing satisfaction scores for 10 questions. Three items showed significant differences after FDR correction: Q5 (weekly reminders helpful, *p* < 0.001), Q6 (app helped improve understanding of asthma, *p* = 0.001), Q7 (would recommend, *p* = 0.018). Overall satisfaction (**lower right**) was significantly higher in the intervention group (*p* = 0.0044). For convenience the significance levels are marked with * for *p* < 0.05 (statistically significant), ** for *p* < 0.01 (highly significant) and with *** for *p* < 0.001 (very highly significant). Multiple testing panels (**lower left**) show the robustness of the findings.

Patient satisfaction with the monitoring system was assessed using a 10-item questionnaire during week 12 (Figure 4). Overall satisfaction was significantly higher in the intervention group (median total score: 9, IQR 7–9) compared to controls (median: 6, IQR 2–8; *p* = 0.0044).

Analysis of individual items revealed three questions with significant differences after false-discovery rate (FDR) correction (Figure 4, middle panel). All intervention patients (100%) reported that weekly reminder messages helped them perform measurements regularly (Q5, *p* < 0.001), whereas 0% of controls who did not receive reminders reported the same. Similarly, 100% of intervention patients agreed that the application helped them better understand their asthma (Q6, *p* = 0.001), compared to 47% of controls. All intervention patients (100%) indicated they would recommend the system to other patients with severe asthma (Q7, *p* = 0.018), compared to 67% of controls. No significant differences were observed for the remaining seven questions (all *p* > 0.05 after FDR correction).

The heatmap (Figure 4, top panel) shows generally high satisfaction scores across both groups, with the intervention group demonstrating consistently higher scores. The multiple testing correction panel (Figure 4, lower left) confirms that the three significant findings (Q5, Q6, Q7) remained robust after controlling for multiple comparisons using the Benjamini–Hochberg FDR procedure.

### 3.7. Safety

No device-related adverse events were reported during the study period. None of the participants experienced adverse respiratory events, injuries, or clinically relevant deterioration attributable to the use of the AioCare system or to the home monitoring procedures. No safety-related discontinuations occurred.

## 4. Discussion

### 4.1. Summary of Main Findings

This prospective pilot study demonstrates that telemedicine-based home monitoring using the AioCare system is feasible in patients with severe asthma and is associated with improved adherence and high patient satisfaction, particularly among those receiving weekly reminder messages. The intervention group showed superior adherence and a consistently positive user experience, indicating strong acceptance of digital home monitoring. No significant improvements in objective lung function were observed over 12 weeks, consistent with the advanced disease stage and optimized therapy of the cohort. A favorable trend toward improved symptom control was identified, although the study was underpowered to detect statistically significant differences. Accordingly, the observed ACT trend should be interpreted with caution and considered hypothesis-generating rather than confirmatory.

Importantly, this study was conceived as a feasibility and adherence-focused pilot trial. Accordingly, the primary value of our findings lies in demonstrating that simple digital reminders can significantly improve adherence to home monitoring in patients with severe asthma, while clinical outcomes were exploratory in nature and the study was not powered to demonstrate clinical efficacy.

### 4.2. Adherence—Primary Success

Asthma is a heterogeneous group of airway disorders marked by chronic inflammation and significant variability in clinical presentation. Outside of exacerbation episodes, numerous patients remain predominantly asymptomatic despite persistent inflammatory activity, significantly complicating long-term adherence to treatment and monitoring. This clinical–biological separation underscores the necessity for interventions engineered to facilitate adherence and prolonged engagement. This technique is relevant to all asthma phenotypes, regardless of inflammatory endotype, and ongoing digital self-monitoring may enable the prompt detection of loss of control and possibly rapidly progressive phenotypes [15,16,17].

The most clinically significant finding was the notable enhancement in adherence among patients who received reminder messages. In severe asthma, where long-term outcomes are significantly influenced by sustained engagement, adherence is a critical behavioral endpoint. The higher completion rate in the intervention group indicates that low-intensity, low-cost reminder strategies can significantly affect patient behavior. These findings align with previous AioCare studies and the wider digital health literature, indicating enhanced adherence through reminder-based telemedicine interventions. Since adherence predicts long-term asthma outcomes, this finding underscores a key potential advantage of telemedicine-supported monitoring [18,19,20].

### 4.3. Educational and Empowerment Benefits

A significant patient-centered outcome was the perceived educational benefit, particularly in the intervention group, in which all patients reported greater awareness of respiratory status and disease comprehension. Regular home spirometry with feedback from an app may help individuals better understand the relationship between symptoms and function and detect early changes in disease control. In addition to education, responses indicated an empowerment effect, leading to increased involvement in self-management. In cases of severe asthma, where getting rid of all the symptoms is often impossible, having the patient feel like they understand and are actively involved in their care are important outcomes.

### 4.4. Why No Functional Improvements Were Observed

The absence of short-term improvement in lung function was expected given the severe, long-standing disease and optimized therapy, including biologics. Baseline spirometry suggested partially fixed airflow limitation, consistent with structural airway changes that were unlikely to reverse over 12 weeks. Furthermore, having current or former smokers in our group may have made it even less likely that people would be able to improve their function. People with asthma who smoke are more likely to have persistent airway inflammation, faster lung function decline, airway remodeling, and a reduced response to corticosteroid therapy. All of these things make airflow obstruction more permanent and less reversible. Telemonitoring primarily influences surveillance, engagement, and early detection rather than biological airway remodeling. Similar findings in severe asthma and COPD telemonitoring studies indicate that digital interventions more consistently affect behavioral and management outcomes than short-term physiological parameters [21,22,23,24,25,26,27,28].

### 4.5. Symptom Trend—Type II Error?

Even though the differences in symptoms between groups were not statistically significant, the medium effect size (d = 0.42) and the higher percentage of participants in the intervention group who met the MCID suggest a clinically important signal. A type II error is possible because we have only about 40% power to detect this effect. These results indicate that we need larger, longer-term trials to determine whether adherence and educational benefits lead to long-term clinical improvement. Consequently, the ACT findings should be viewed as preliminary and exploratory, rather than as evidence of clinical efficacy.

### 4.6. Patient Satisfaction as a Key Finding

Patients were always willing to try the intervention, and everyone in the intervention group agreed that reminders and educational value were important. Patients valued involvement, reassurance, and perceived supervision even though they didn’t see any short-term functional gains. This incident underscores the importance of evaluating telemedicine not only by its physical effects but also by its capacity to enhance the patient experience, patient involvement, and long-term care partnerships.

### 4.7. Comparison to Literature

Few randomized controlled trials of telemonitoring exist for severe asthma. Studies currently available, as well as recent reviews, indicate that study designs differ, and effects on significant clinical endpoints are not always consistent. However, the effects on behavioral and engagement outcomes are more consistent. Evidence from COPD telemonitoring indicates inconsistent physiological outcomes but consistent advantages in adherence and self-management. Our group stands out from other digital asthma studies because everyone was thrilled with the program and could clearly see how it helped them learn. Previous AioCare studies validate the viability and acceptability of this monitoring methodology [18,26,29,30,31,32,33].

### 4.8. Clinical Implications

Telemedicine-based home monitoring may provide clinically significant advantages beyond transient alterations in lung function. Self-monitoring may help patients with severe asthma remain engaged, learn more about their condition, and manage it proactively, particularly when they are motivated. Although the long-term effects weren’t examined, the observed benefits of adherence and satisfaction warrant further research. The amount of money these behavioral changes save will depend on whether they lead to fewer flare-ups and reduced use of healthcare services.

### 4.9. Strengths

The randomized controlled design, focus on severe asthma, comprehensive patient-centered outcomes, high follow-up completion, use of validated ACT measures, and a strict statistical framework that includes intention-to-treat and longitudinal modeling are all important strengths.

### 4.10. Limitations

Although the small sample size limits generalizability and reduces statistical power, the effect-size estimates indicates the need for further research. Post hoc analyses confirmed that the study was underpowered to detect small changes in lung function, but adequately powered for adherence and satisfaction outcomes, supporting the feasibility focus of this pilot trial.

The open-label design represents an important limitation of this study. Because both patients and investigators were aware of group allocation, adherence behavior and satisfaction ratings may have been influenced by expectation effects and increased attention associated with receiving reminders (performance and reporting bias), including a potential Hawthorne effect, whereby participants may have been more compliant simply because they were aware of being monitored as part of a study. Consequently, the observed improvements in adherence and user experience should be interpreted with caution.

In addition, although the satisfaction questionnaire was adapted from a validated instrument (MAUQ), its modification into a dichotomous yes/no format and the inclusion of supplementary items may have weakened its psychometric validity and limited comparability with other studies. Therefore, the satisfaction findings should be interpreted as exploratory and supportive rather than definitive evidence.

The 12-week follow-up was useful for assessing feasibility and adherence, but it was insufficiently long to evaluate long-term clinical endpoints. The study’s generalizability to other settings is limited by its single-center design and the absence of a cost-effectiveness analysis. Another limitation is that the study was not registered prospectively before enrollment. The trial was registered retrospectively at ClinicalTrials.gov (Identifier: NCT07393984) to promote transparency and adhere to current reporting standards. Although retrospective registration does not fully align with best practices for clinical trial conduct, all study procedures, outcomes, and analyses were outlined beforehand. Finally, the comprehensive statistical approach enabled thorough exploration of multiple outcomes and longitudinal patterns. However, future confirmatory trials should adopt more focused analytical plans, prioritizing prespecified primary endpoints with streamlined statistical methods to maximize clarity and clinical utility.

### 4.11. Future Directions

Future studies should include larger multicenter randomized trials with 6- to 12-month follow-up. These trials should focus on flare-ups and long-term control. To improve telemedicine-supported monitoring strategies for severe asthma, it will be important to perform subgroup analyses, health-economic evaluations, and qualitative patient research.

## 5. Conclusions

This randomized controlled trial demonstrates the feasibility and potential benefits of telemedicine-based home monitoring using the AioCare system in patients with severe asthma.

Weekly reminder messages significantly enhanced adherence to home spirometry monitoring compared to standard monitoring without reminders. Both groups showed a favorable trend toward improved asthma symptom control, as measured by the Asthma Control Test (ACT), although no statistically significant between-group differences were observed. No changes in objective lung function parameters were detected over the 12-week period, which is consistent with the advanced disease stage and optimized biologic therapy in the study population. Patient satisfaction was high overall, with notably higher scores in the intervention group, particularly regarding the utility of reminders, improved understanding of the disease, and willingness to recommend the system.

The intervention group achieved superior adherence (mean 11.47 ± 0.92 sessions vs. 9.13 ± 3.16 in controls; *p* = 0.044) and greater overall satisfaction (median score 9 vs. 6; *p* = 0.0044). A favorable directional trend in symptom control was observed in both groups, with a medium effect size for the between-group difference (Cohen’s d = 0.42). The study was underpowered to detect small effects on lung function but adequately powered for adherence and satisfaction outcomes. No adverse events were reported, confirming the safety of the approach.

Digital self-monitoring with simple, low-cost reminders represents a promising adjunct to biologic therapy in severe asthma, fostering better patient engagement, adherence, and disease awareness without requiring intensive clinical resources. This patient-centered strategy may support proactive management and empowerment, particularly in real-world settings where traditional follow-up is limited.

Future research should involve larger multicenter trials with extended follow-up (at least 6–12 months) to evaluate long-term clinical outcomes, including exacerbation rates, healthcare utilization, and cost-effectiveness, and to determine whether the demonstrated engagement and adherence benefits translate into meaningful long-term clinical improvements. Subgroup analyses could identify patients who benefit most, while qualitative studies may refine engagement strategies for broader implementation.

## Figures and Tables

**Table 1:** Baseline Characteristics.

Characteristic	Control (*n* = 15)	Intervention (*n* = 15)	*p*-Value
**Demographics**			
Age, years	52.3 ± 12.0	52.3 ± 12.9	0.988
Female sex, n (%)	10 (66.7)	6 (40.0)	0.272
BMI, kg/m^2^	30.0 ± 4.4	27.7 ± 3.0	0.109
**Smoking History**			
Current smoker, n (%)	8 (53.3)	5 (33.3)	0.461
Former smoker, n (%)	0 (0.0)	1 (6.7)	1
**Baseline Lung Function**			
FVC, L	3.61 ± 1.03	3.68 ± 1.18	0.877
FVC, % predicted	92.5 ± 20.6	88.0 ± 23.2	0.596
FEV1‚ L	2.57 ± 0.91	2.65 ± 1.00	0.816
FEV1‚ % predicted	81.2 ± 21.5	78.0 ± 23.1	0.717
FEV1/FVC ratio	69.92 ± 9.60	70.93 ± 10.85	0.801
**Baseline Symptom Control**			
ACT score	18.7 ± 1.9	19.0 ± 2.0	0.642
Well controlled, n (%)	5 (33.3)	6 (40.0)	
Partly controlled, n (%)	10 (66.7)	8 (53.3)	
Poorly controlled, n (%)	0 (0.0)	1 (6.7)	
**Medications**			
Biological therapy, n (%)	15 (100.0)	15 (100.0)	1

**Table 2:** ACT Scores and Clinical Significance.

Measure	Control (*n* = 15)	Intervention (*n* = 15)	Observations
**Baseline ACT score**	18.67 ± 1.91	19.00 ± 1.96	Groups balanced (*p* = 0.641)
**Week 12 ACT score**	21.40 ± 1.59	22.40 ± 1.59	-
**Change from baseline**	2.73 ± 1.58	3.40 ± 1.59	-
**Within-group *p*-value**	t = −6.70, *p* < 0.001	t = −8.26, *p* < 0.001	Both significant

Note: Data presented as mean ± SD. Change scores calculated as Week 12—Baseline. Within-group changes were assessed by paired *t*-tests. Between-group comparison by independent *t*-test on change scores (*t* = −1.15, *p* = 0.260, Cohen’s d = 0.42). Minimal clinically important difference (MCID) = 3 points; proportion achieving MCID compared by chi-square test (χ^2^ = 0.139, *p* = 0.709). ACT = Asthma Control Test.

## Data Availability

The data presented in this study are available on request from the corresponding author.

## Supplementary Materials

The following supporting information can be downloaded at: https://www.mdpi.com/article/10.3390/medicina62020368/s1, Supplementary Methods: Detailed Statistical Analysis; Supplementary Table S1: Longitudinal Lung Function Outcomes; Supplementary Results: Missing Data and Post-Hoc Power.

## Abbreviations

The following abbreviations are used in this manuscript:

ACT	Asthma Control Test
TSLP	Thymic Stromal Lymphopoietin
GINA	Global Initiative for Asthma
ATS	American Thoracic Society
ERS	European Respiratory Society
BMI	Body Mass Index
PEF	Peak Expiratory Flow
SpO_2_	Oxygen Saturation
GEEs	Generalized Estimating Equations
GenAI	Generative artificial intelligence
SMS	Short Message Service
MCAR	Missing Completely At Random
MAR	Missing At Random
COPD	Chronic Obstructive Pulmonary Disease
MCID	Minimum Clinically Relevant Difference
FEF_50%_	Forced Expiratory Flow at 50% of Forced Vital Capacity
FEV1	Forced Expiratory Volume in the First Second
FVC	Forced Vital capacity
