# Supplementary material for: Effect of Digital Self-Monitoring on Patient Engagement and Clinical Outcomes in Severe Asthma: A Randomized Controlled Pilot Study
Source: Medicina (Kaunas). 2026 Feb 12;62(2):368. doi: 10.3390/medicina62020368 (PMC12941848; doi:10.3390/medicina62020368)
Supplement: Supplementary file 1 [file medicina-62-00368-s001.zip › medicina-4123692-supplementary.pdf]

Supplementary Materials

Effect of Digital Self-Monitoring on Patient Engagement and Clinical Outcomes in Severe Asthma: A Randomized Controlled Pilot Study

Wellmann N, Ancusa VM, Marc MS, Trusculescu AA, et al.

Supplementary Methods. Detailed Statistical Analysis

Statistical analyses were performed using Python version 3.12.12, employing the following libraries: Pandas (v2.2.2), SciPy (v1.16.3), Statsmodels (v0.14.6), Scikit-learn (v1.6.1), NumPy (v2.0.2), Matplotlib (v3.10.0), and Seaborn (v0.13.2). All statistical tests were two-tailed, with a predefined significance threshold of $\alpha = 0.05$.

The primary analysis followed the intention-to-treat principle and included all randomized participants in their originally assigned groups, irrespective of protocol adherence or study completion. Longitudinal outcomes, including spirometry parameters and Asthma Control Test (ACT) scores, were analyzed using linear mixed-effects models (LMMs) to account for within-subject correlations across repeated weekly measurements. In these models, participants were treated as random effects through random intercepts, while fixed effects included study group (intervention versus control), time (weeks 1–12), and the interaction between group and time. The group-by-time interaction term was considered the primary parameter of interest, with a statistically significant interaction indicating divergent outcome trajectories between groups over the study period.

Model assumptions were assessed through Shapiro–Wilk tests for normality of residuals and by visual inspection of residual diagnostic plots, including Q–Q plots and residuals versus fitted values. In cases where normality assumptions were violated, generalized estimating equations (GEEs) with a Gaussian family and an exchangeable correlation structure were applied as a robust alternative, providing population-averaged estimates.

Secondary analyses focused on cross-sectional comparisons between groups. Baseline characteristics, overall adherence rates, and satisfaction questionnaire scores were compared using independent-samples t-tests for normally distributed continuous variables and Mann–Whitney U tests for non-normally distributed or ordinal variables. Categorical variables were analyzed using chi-square tests. Effect sizes were reported as Cohen's d for parametric comparisons and rank-biserial correlation coefficients for non-parametric analyses. For the 10-item AioCare Satisfaction Questionnaire, adjustment for multiple testing was performed using the Benjamini–Hochberg false discovery rate procedure, with a corrected significance level of $\alpha = 0.05$.

Patterns of missing data were evaluated descriptively and examined for associations with baseline characteristics. Linear mixed-effects models inherently accommodate missing observations under the missing-at-random assumption and therefore did not require imputation for the primary analyses. Post-hoc power analyses were conducted using F-test ANOVA-based calculations to estimate achieved statistical power for observed effect sizes and to determine the minimum detectable effect sizes corresponding to 80% power.

Supplementary Table S1. Longitudinal Lung Function Outcomes

Outcome	Control Baseline	Intervention Baseline	Control Week 12	Intervention Week 12	Control Change	Intervention Change	Group× Time β	95% CI	P-value
FVC, L	3.61 ± 1.03	3.68 ± 1.18	3.42 ± 1.24	3.99 ± 1.17	0.01 ± 0.34	0.09 ± 0.29	0.0054	(-0.0151, 0.0259)	0.605
FVC, % predicted	92.5 ± 20.6	88.0 ± 23.2	90.5 ± 20.3	94.7 ± 21.3	0.2 ± 8.6	2.1 ± 8.0	0.1246	(-0.4411, 0.6902)	0.666
FEV_1, L	2.57 ± 0.91	2.65 ± 1.00	2.45 ± 1.04	2.88 ± 0.86	0.06 ± 0.26	0.02 ± 0.26	0.0003	(-0.0157, 0.0163)	0.971
FEV_1, % predicted	81.2 ± 21.5	78.0 ± 23.1	81.2 ± 24.0	87.2 ± 19.6	3.1 ± 10.3	2.9 ± 10.8	0.0849	(-0.5132, 0.6829)	0.781
$FEF_{25\text{–}75}$, L/s	1.93 ± 1.03	2.12 ± 1.14	1.82 ± 1.02	2.33 ± 0.83	0.16 ± 0.36	0.06 ± 0.46	-0.0089	(-0.0354, 0.0175)	0.509
$FEF_{25\text{–}75}$, % predicted	65.8 ± 20.7	69.5 ± 20.2	63.2 ± 29.5	72.7 ± 21.0	2.8 ± 16.5	-5.3 ± 15.5	-0.3601	(-1.3527, 0.6325)	0.477
PEF, L/s	5.56 ± 2.53	7.10 ± 3.11	5.27 ± 2.25	7.29 ± 2.78	0.16 ± 0.42	-0.21 ± 0.52	-0.0145	(-0.0488, 0.0198)	0.407
PEF, % predicted	80.5 ± 20.5	89.6 ± 7.2	88.0 ± 13.7	84.8 ± 12.7	4.3 ± 6.8	-1.5 ± 6.2	-0.2921	(-0.7201, 0.1360)	0.181

Table S1. Primary Outcomes. Note: Data are presented as mean ± SD. Change scores calculated as Week 12 - Baseline. Group×Time interaction coefficient (β) from generalized estimating equations (GEEs) models represents differential change rate between groups over 12 weeks. GEE was used for all outcomes due to violation of normality assumptions (Shapiro-Wilk $p<0.05$ for all outcomes). Negative β indicates greater decline in intervention group; positive β indicates greater improvement in intervention group. All 95% confidence intervals include zero. FVC = forced vital capacity; FEV_1 = forced expiratory volume in 1 second; $FEF_{25\text{–}75}$ = forced expiratory flow at 25-75%; PEF = peak expiratory flow.

Supplementary Results. Missing Data and Post-Hoc Power

Missing data for spirometry outcomes ranged from 14% to 15% across timepoints, primarily due to technical difficulties during measurements or scheduling conflicts. Little's test for missing completely at random (MCAR) was non-significant ($\chi^2=142.3$, df=156, p=0.784), indicating that missingness was unrelated to observed or unobserved variables. Mixed-effects models, which use maximum likelihood estimation to handle missing data under the missing at random (MAR) assumption, were therefore appropriate for analysis.

Post-hoc power analysis revealed that the study was underpowered to detect the small lung function effects observed (Group×Time interaction effect sizes $f^2<0.01$), with achieved power <50% for all spirometry outcomes. In contrast, adequate power (>80%) was achieved for adherence (r=0.40,

observed power=82%) and overall satisfaction (d=0.99, observed power>95%) outcomes, where medium to large effect sizes were observed.
